# Supplementary material for: Differential Pd-nanocrystal facets demonstrate distinct antibacterial activity against Gram-positive and Gram-negative bacteria
Source: Nat Commun. 2018 Jan 9;9:129. doi: 10.1038/s41467-017-02502-3 (PMC5760645; doi:10.1038/s41467-017-02502-3)
Supplement: Supplementary file 1 — Supplementary Information [file 41467_2017_2502_MOESM1_ESM.pdf]

Supplementary Methods

Characterization of palladium nanocrystals. Powder X-ray diffraction patterns (XRD) were obtained by using a X-ray diffractometer (D8 advance, Bruker, German) and data were collected from 5° to 90° with Cu-Kα (λ = 1.54056 Å) radiation. X-ray photoelectron spectroscopy (XPS) were measured on an SSI S-Probe XPS Spectrometer (ESCALAB 250Xi, Thermo Scientific, USA). The Pd^0 peak at 335.0 eV was used as a reference to correct for charging effects. Fourier transformation infrared (FTIR) spectra were conducted in the range 3500-500 cm^{-1} on a FTIR spectrometer (Nicolet iS50, Thermo Scientific, USA) in the transmission mode. Thermogravimetric analyses (TGA) were accomplished on a simultaneous thermogravimetric analyzer (STA 449F, NETZSCH, German) in the temperature range from 30 to 800 °C under a nitrogen flow with the heating rate of 10 °C min^{-1}.

Dissolution experiments. To evalute the release of Pd^{2+} from Pd nanocrystals, 1 ml of different concentrations of Pd nanocrystals (900 μg/ml for Pd cubes and 310 μg/ml for Pd octahedrons) were placed in dialysis bags (MWCO, 2000 Da) and dialyzed against 20 ml of H_2O at 37 °C with constant shaking, respectively. Then, 5 ml of solution containing release Pd^{2+} was taken out and replaced by 5 ml fresh H_2O to maintain submersed conditions at desired time intervals. The concentration of released Pd^{2+} solution was measured with an inductively-coupled plasma mass spectrometry (ICP-MS, ELEMENT 2, Thermo, USA). Three replicates of each sample were run for statistical purposes.

Supplementary Figures

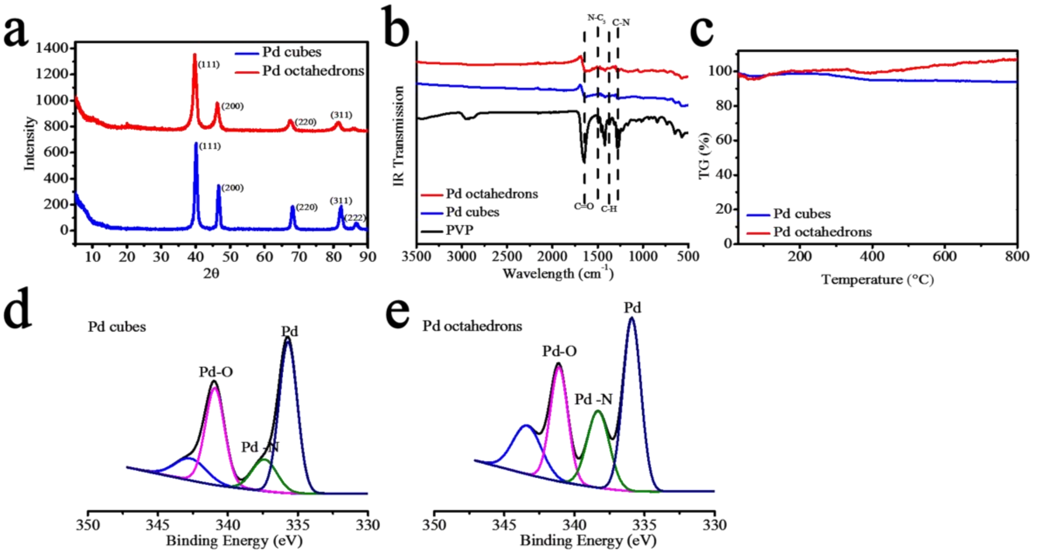

Supplementary Figure 1. Characterization of the Pd nanocrystals. (a) XRD pattern (b) FITR spectra (c) TGA curves (d, e) XPS spectra of the cubic and octahedral Pd nanocrystals, respectively.

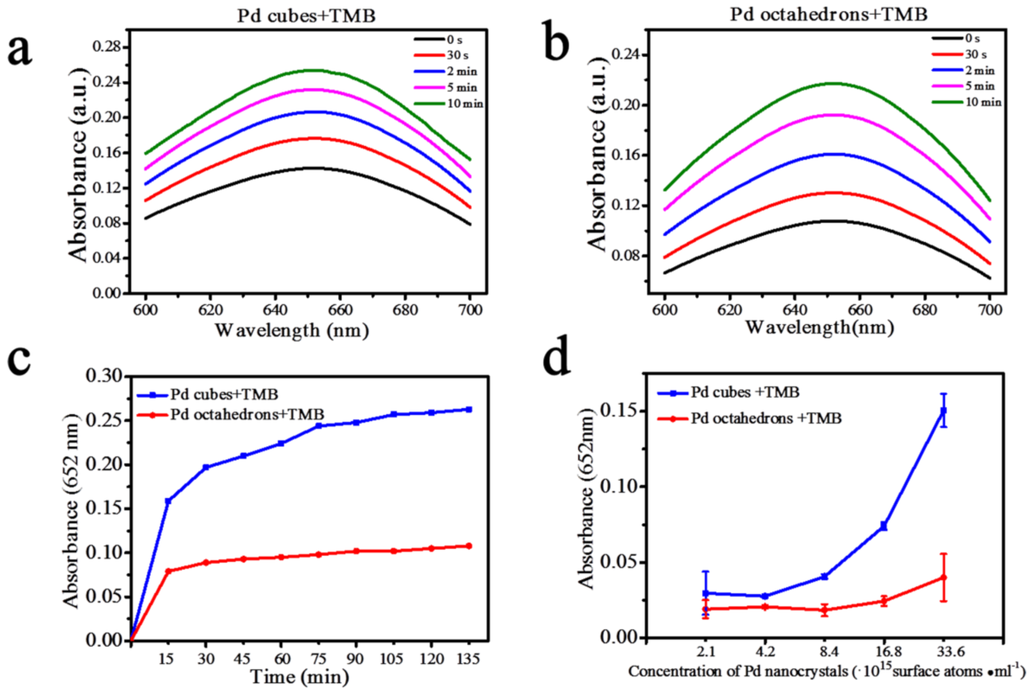

Supplementary Figure 2. The absorbance spectra of TMB in different reaction systems: (a) Pd cubes+TMB and (b) Pd octahedrons+TMB; (c) Time-dependent absorbance changes at 652 nm of TMB in different reaction systems; (d) Concentration-dependent absorbance changes at 652 nm of TMB in different reaction systems. Data are presented as means ± standard deviation (s.d.). Error bars were based on s.d. of three independent experiments.

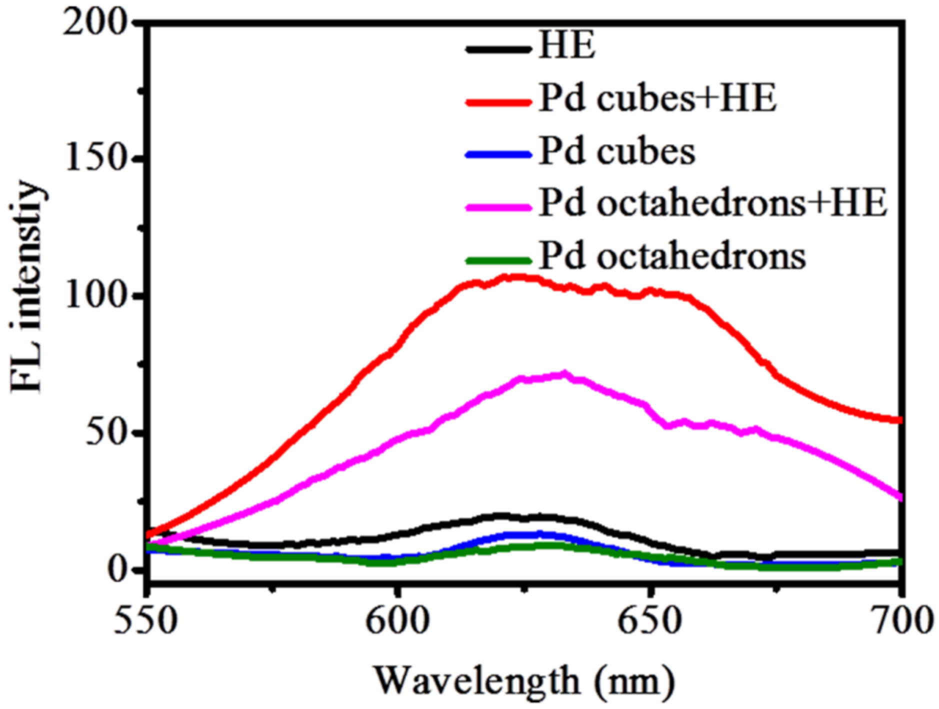

Supplementary Figure 3. Fluorescence spectra of hydroethidine (HE) before and after the addition of Pd nanocrystals.

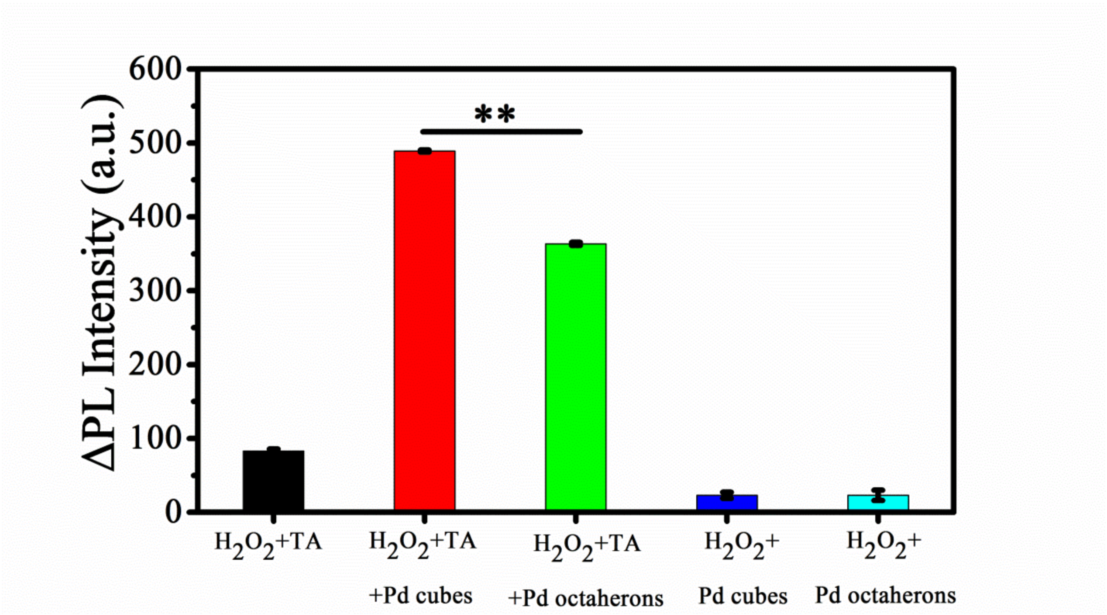

Supplementary Figure 4. Histograms of the ΔPL intensity show the fluorescence product, 2-hydroxy terephthalic acid (TAOH), catalyzed by the Pd nanocrystals; Data are presented as means ± s.d.. Error bars were taken from three independent experiments. *P* values were calculated by the student's test: $^{**}p < 0.01$.

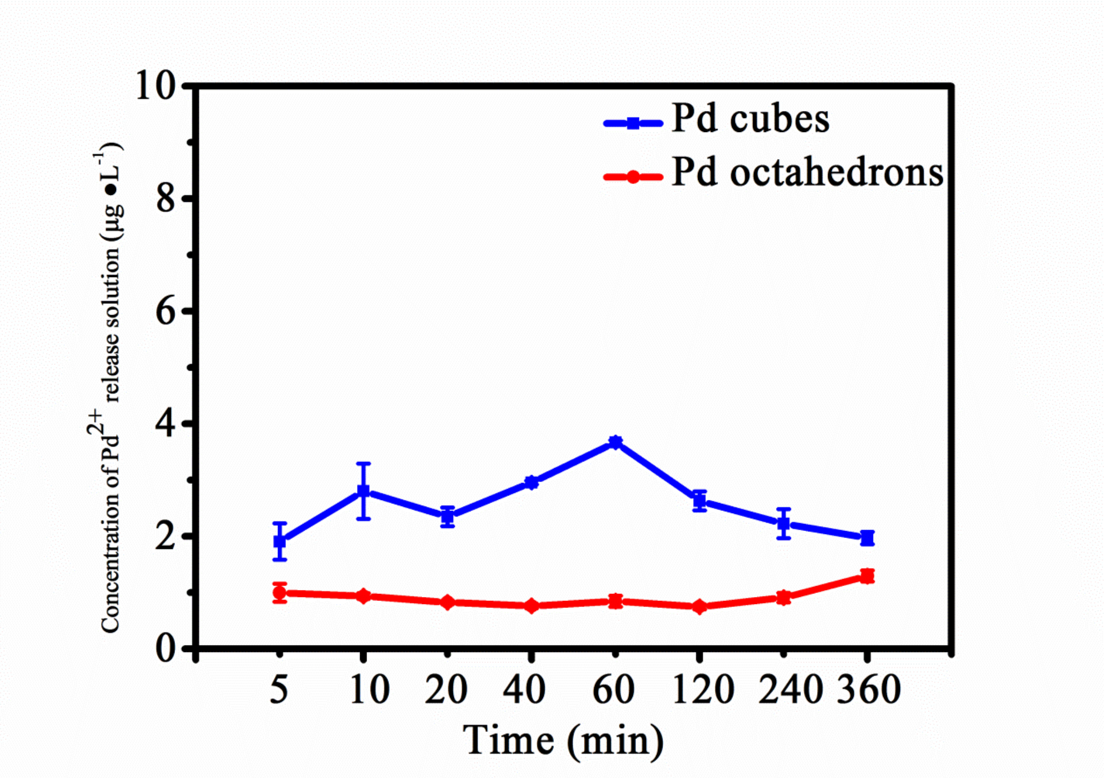

Supplementary Figure 5. Profiles for Pd^{2+} release from the Pd nanocrystals. Data are expressed as means ± s.d. (n=3).

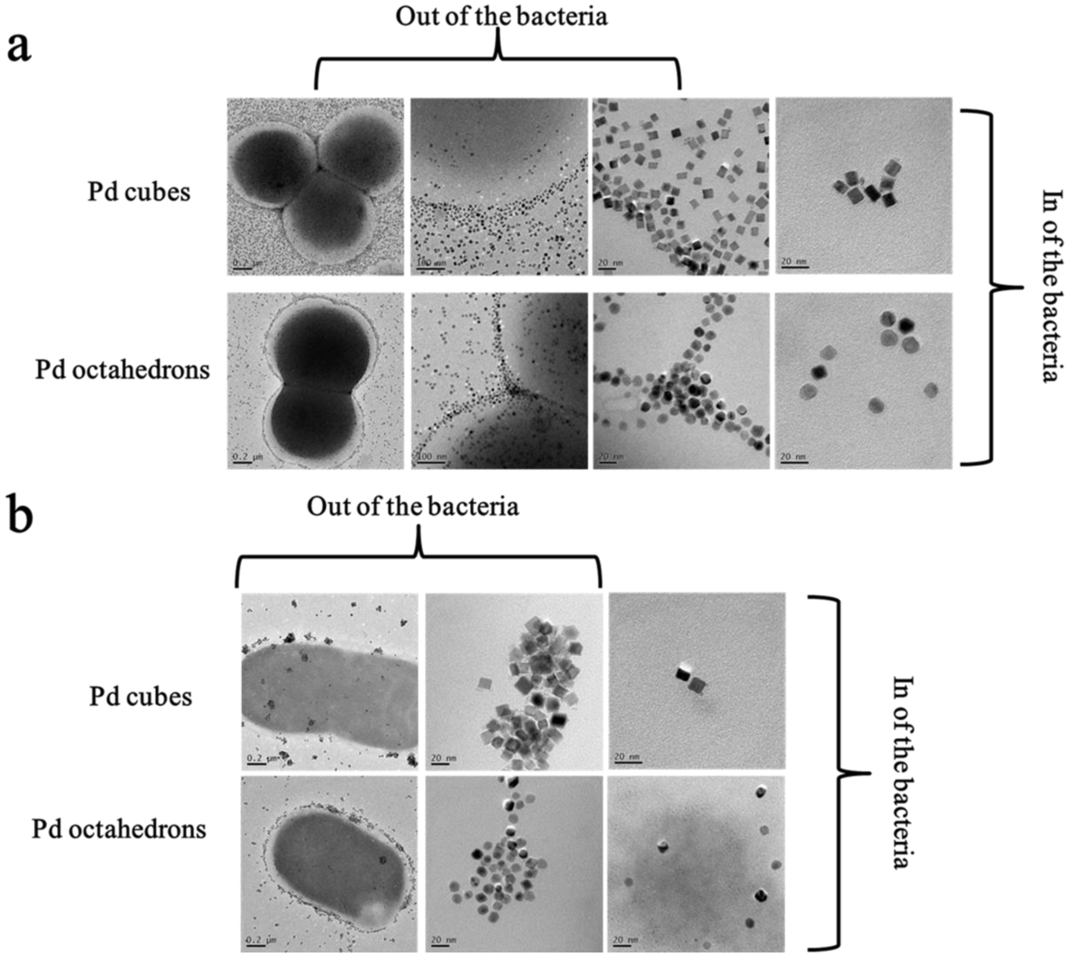

Supplementary Figure 6. The TEM characterization of the Pd nanocrystals inside and outside for the: (a) Gram-positive *S. aureus* and (b) Gram-negative *E. coli* bacteria.

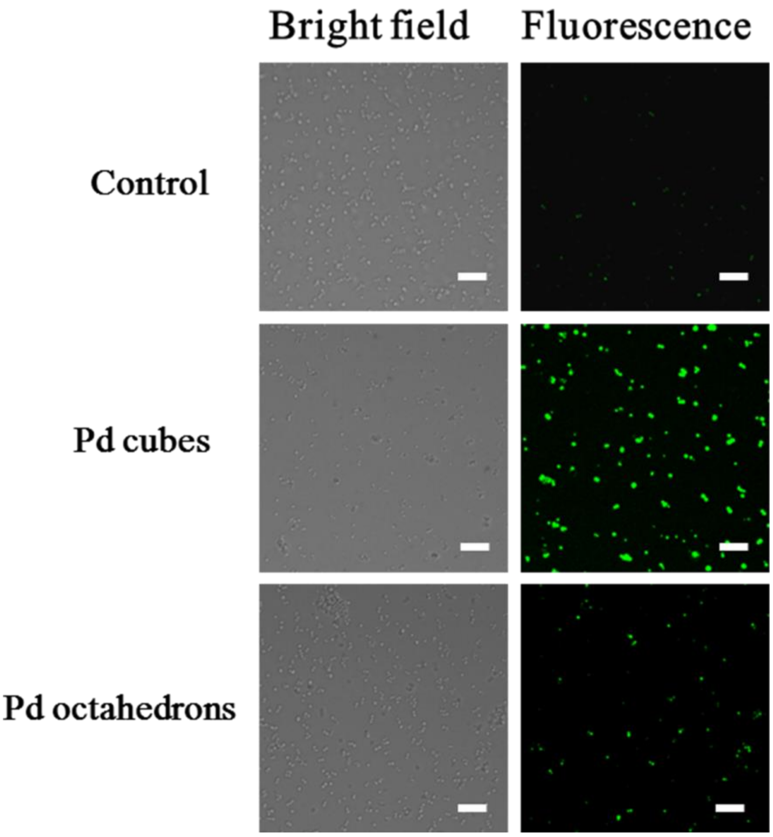

Supplementary Figure 7. Evaluation of ROS content measured as CM-DCF fluorescence. The bright field and fluorescence micrographs of the *S. aureus* cells after various treatments. Scale bar: 10 μm.

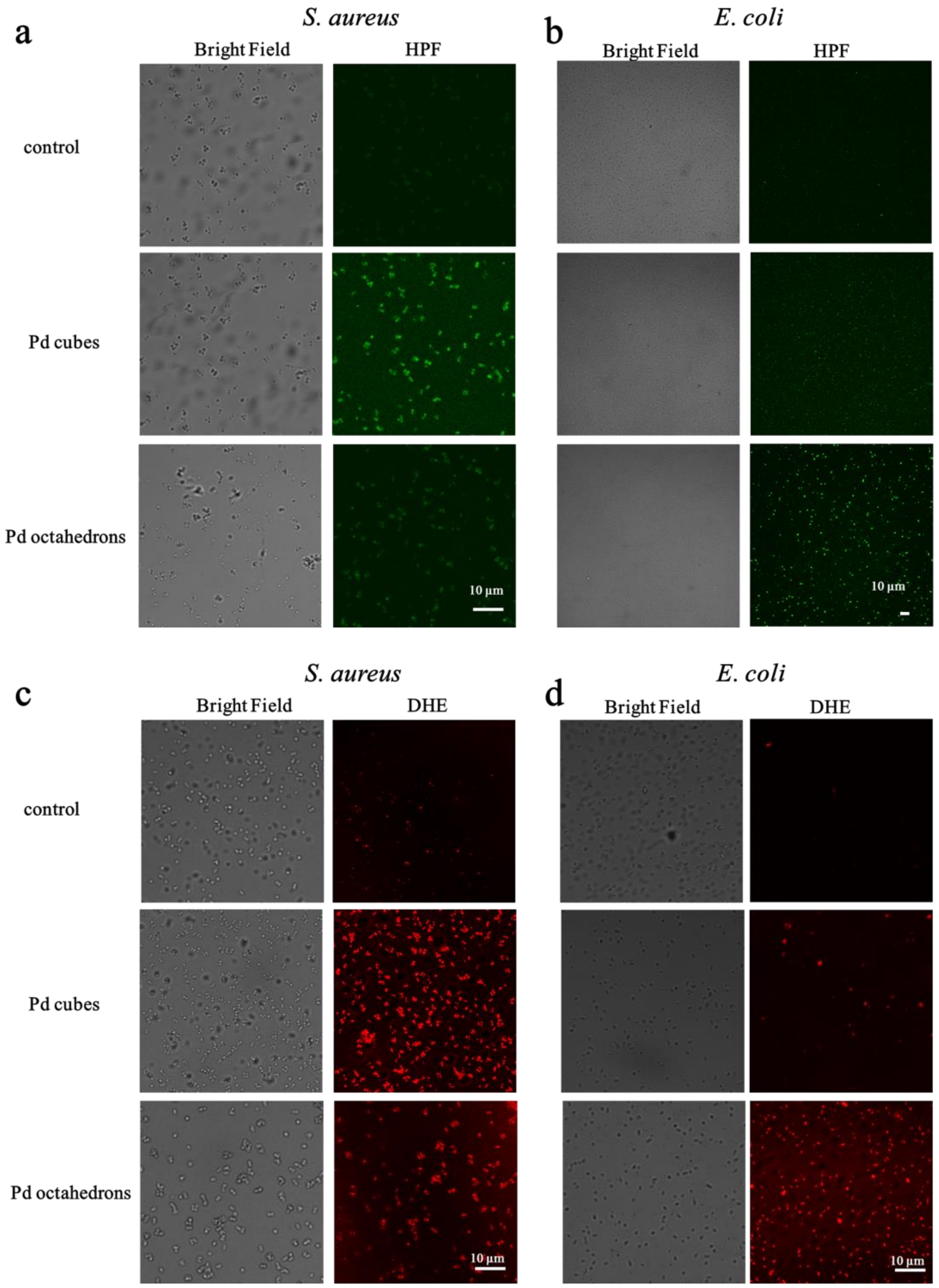

Supplementary Figure 8. (a, b) Evaluation of hydroxyl radical content, measured as HPF fluorescence probes. The bright field and fluorescence micrographs of bacterial after various treatments. (a) *S. aureus* (b) *E. coli*; (c, d) Evaluation of superoxide radical content, measured as DHE fluorescence probes. The bright field and fluorescence micrographs of bacterial after various treatments. (c) *S. aureus* (d) *E. coli*.

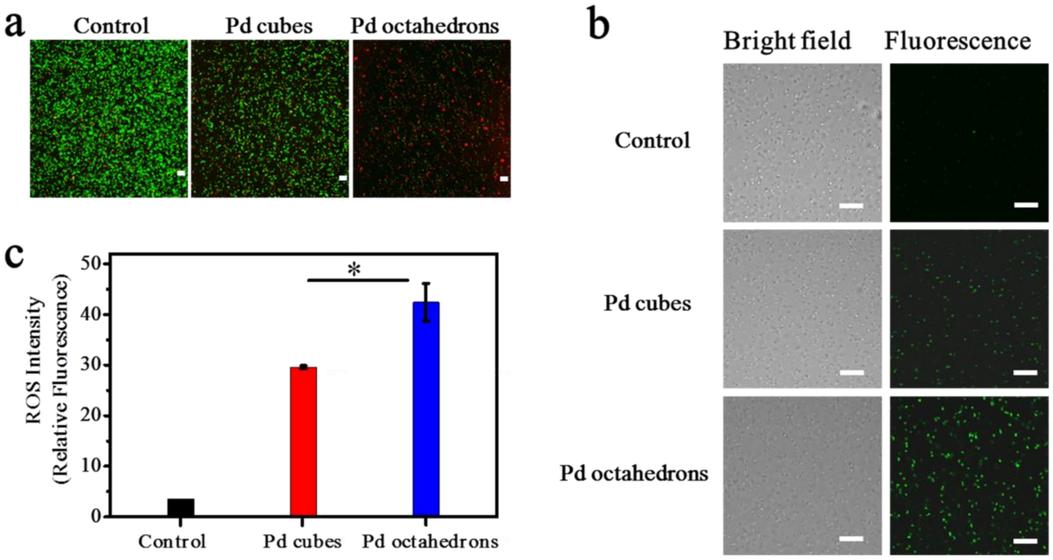

Supplementary Figure 9. (a) Representative fluorescence images of live (green) and dead (red) *E. coli* cells after various treatments. Scale bar: 10 μm. (b) Evaluation of ROS content measured as CM-DCF fluorescence. The bright field and fluorescence micrographs of the *E. coli* cells after various treatments. Scale bar: 10 μm. (c) Quantitative analysis of the ROS levels in bacterial cells for the control group and in the presence of the different Pd nanocrystals. Data is represented as the mean fluorescence intensity and standard deviation from three regions. *P* values were calculated by the student's test: $*p < 0.05$.

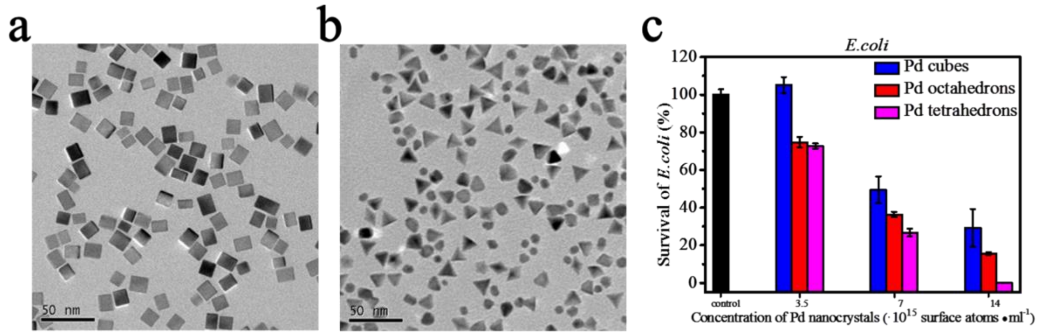

Supplementary Figure 10. (a, b) TEM images of the Pd cubes and Pd tetrahedrons. (c) Antibacterial activity of the Pd nanocrystals against the gram-negative *E. coli* bacteria. Data are presented as means ± s.d. (n = 3).

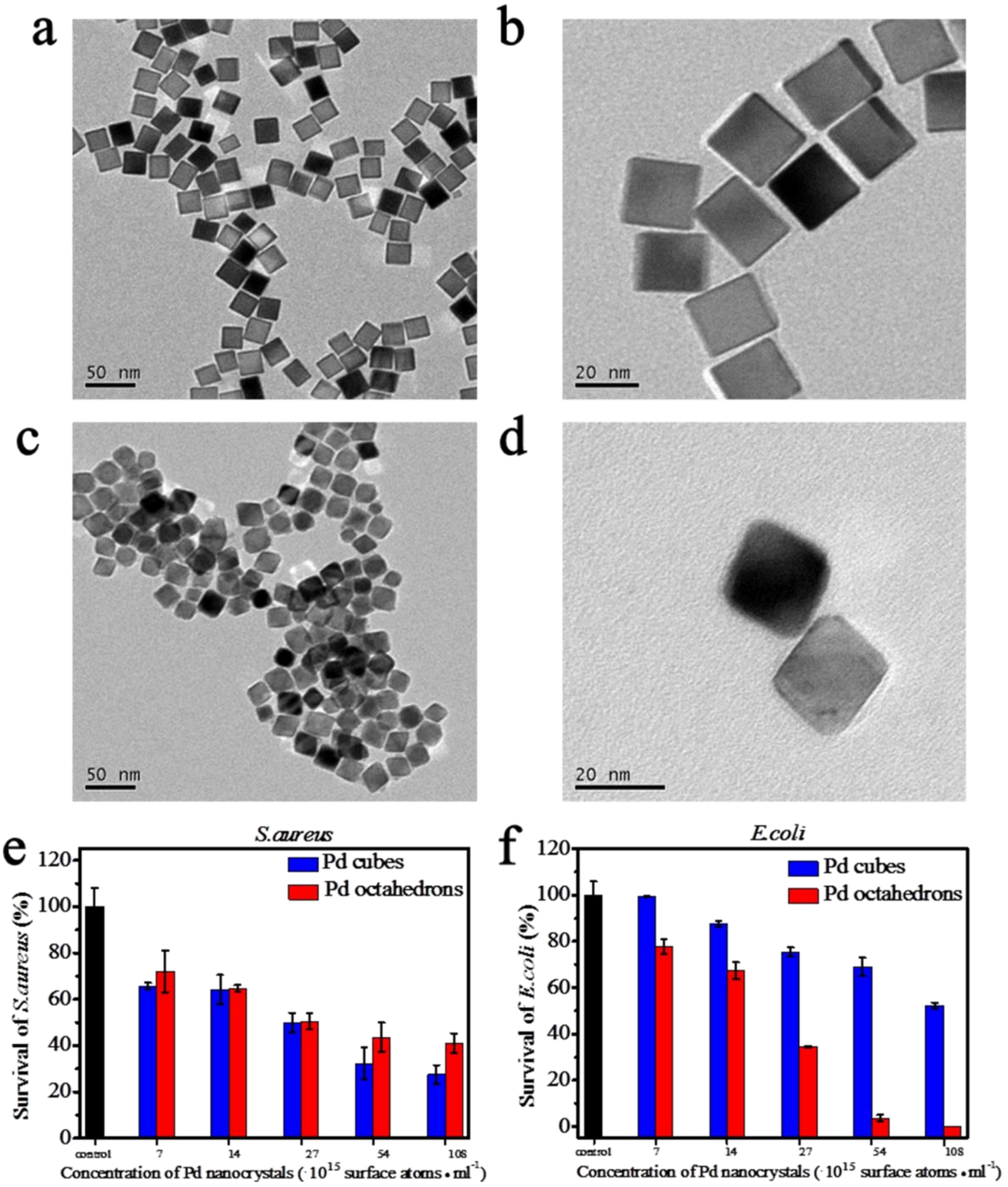

Supplementary Figure 11. (a, b) TEM images of large-sized Pd cubes (c, d) TEM images of large-sized Pd octahedrons (e, f) Antibacterial activity of the large-sized Pd nanocrystals against *S. aureus* and *E. coli* bacteria. Data are presented as means ± standard deviation (s.d.) (n=3).

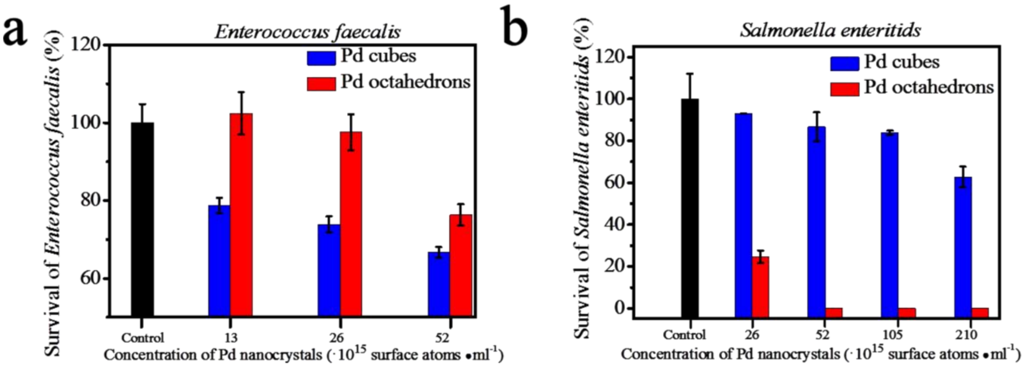

Supplementary Figure 12. Distinct antibacterial activity of the Pd nanocrystals against (a) the Gram-positive *Enterococcus faecalis* and (b) the Gram-negative *Salmonella enteritidis* bacteria. Data are presented as means ± standard deviation (s.d.) (n=3)

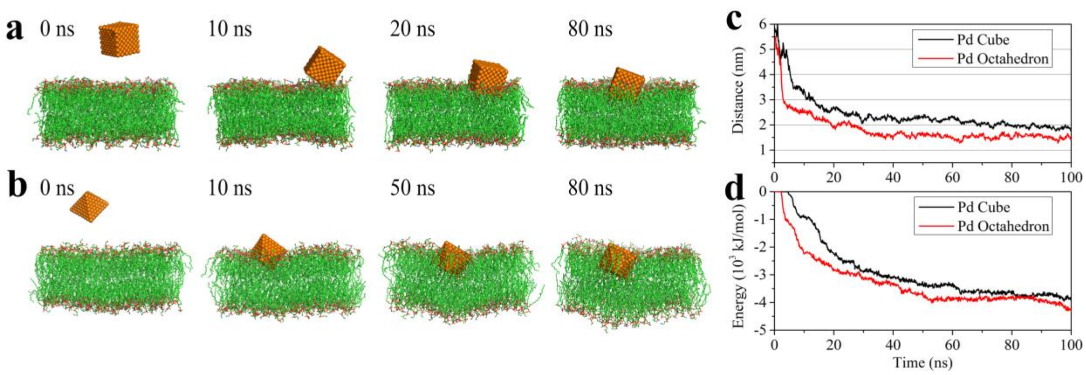

Supplementary Figure 13. Representative snapshots for the membrane penetration event of (a) the Pd cube and (b) the Pd octahedron. Since the membrane penetration process is more complete, more mechanistic distinctions in the interaction of the Pd nanocrystals and the bacterial-mimetic membrane are observed. (c) Time evolution of the distance between the center of mass of the membrane and the two Pd nanoparticles; (d) Time evolution of the interaction energy between the membrane and the two Pd nanoparticles (here the pulling force constant is k_L=200 kJ·mol^{-1}·nm^{-1}).

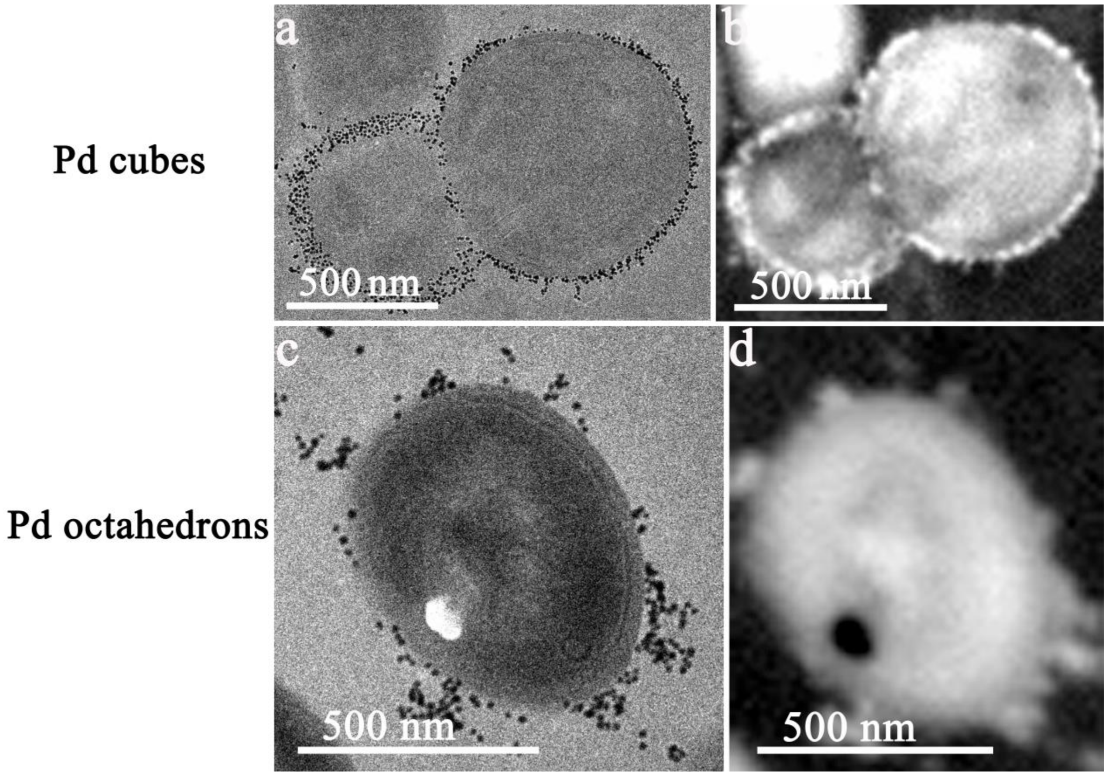

Supplementary Figure 14. TEM (a, c) and STEM (b, d) micrographs of *S. aureus* cells exposed to different Pd nanocrystals show the accumulation of Pd nanoparticles in the vicinity of the bacterial cell wall.
